# Poor prognosis of nucleophosmin overexpression in solid tumors: a meta-analysis

**DOI:** 10.1186/s12885-018-4718-6

**Published:** 2018-08-20

**Authors:** Siying Chen, Hairong He, Yan Wang, Leichao Liu, Yang Liu, Haisheng You, Yalin Dong, Jun Lyu

**Affiliations:** 1 Department of Pharmacy, the First Affiliated Hospital of Xi’an Jiaotong University, No. 277 of Yanta west road, Xi’an, 710061 Shaanxi China; 2 Clinical Research Center, the First Affiliated Hospital of Xi’an Jiaotong University, No. 277 of Yanta west road, Xi’an, 710061 Shaanxi China

**Keywords:** NPM, Sold tumors, Prognosis, Meta-analysis

## Abstract

**Background:**

Nucleophosmin is a non-ribosomal nucleolar phosphoprotein that is found primarily in the nucleolus region of cell nucleus, plays multiple important roles in tumor processes. Accumulated previous studies have reported a potential value of NPM acted as a biomarker for prognosis in various solid tumors, but the results were more inconsistency. We performed this meta-analysis to precisely evaluate the prognostic significance of NPM in solid tumors.

**Methods:**

Clinical data were collected from a comprehensive literature search in PubMed, Web of Science, Embase, and China National Knowledge Infrastructure databases (up to October, 2017). A total of 11 studied with 997 patients were used to assess the association of NPM expression and patients’ overall survival (OS). The hazard ratio (HR) or odds ratio (OR) with its 95% confidence intervals (CI) were calculated to estimate the effect.

**Results:**

The pooled results indicated that higher expression of NPM was observably correlated with poor OS in solid tumor (HR = 1.85, 95% CI: 1.44–2.38, *P* < 0.001). Furthermore, high expression of NPM was associated with some phenotypes of tumor aggressiveness, such as tumor stage (4 studies, III/IV vs. I/II, OR = 5.21, 95% CI: 2.72–9.56, *P* < 0.001), differentiation grade (poor vs. well/moderate, OR = 1.82, 95% CI: 1.01–3.27, *P* = 0.046).

**Conclusion:**

This meta-analysis indicated that NPM may act as a valuable prognosis biomarker and a potential therapeutic target in human solid tumors.

## Background

Nucleophosmin (NPM), also known as B23, numatrin or NO38, was originally identified as a nucleolar phosphoprotein [1]. It was abundantly expressed in the granular region of the nucleolus, which could shuttle between the nucleus and cytoplasm during the cell cycle [2]. NPM consists of 294 amino acids [3]. It is highly conserved phosphoprotein and extensively distributed among different species. Its molecular weight is around 37 kDa and isoelectric point (pI) is 5.1 to 5 [4].

NPM is a multifunctional nucleolar phosphoprotein. Previous studies showed that NPM acted as a factor in ribosome biogenesis, which could regulate ribosome assembly and transport ribosomal proteins to the cytoplasm [5]. Additionally, it was proposed that NPM possessed molecular chaperone activities, such as preventing protein aggregation, preserving enzymes activities during thermal denaturation of several different proteins and facilitating renaturation of chemically-denatured proteins [6]. Recently, several studies suggested that NPM played a crucial role in cell growth, proliferation and transformation. It could regulate cell cycle progression and centrosome duplication [7, 8]. NPM was able to regulate the activity and stability of crucial tumor suppressors such as p53 and ARF [9]. NPM also participated in transcription activation by interacting with transcription factors NF-κB and c-Myc [10, 11].

In addition, numerous studies displayed NPM could be involved in tumorgenesis. Although NPM is frequently mutated in acute myeloid leukemias [12], it is higher expression in many types of human solid tumors, and it has been proposed as a marker for colon, liver, stomach, ovary, thyroid and prostate carcinoma [3, 13–17]. In some cases, because NPM binds to linker histone H1.5, enforced expression of NPM could suppress apoptosis in H1.5 depleted glioma cells, it suggested that effectiveness of targeting NPM could be a potential treatment for glioblastoma [18]. Overexpression of NPM may intensively influence the effects of estrogen on the malignant progression of endometrioid adenocarcinoma via ERα signaling [19, 20]. On the contrary, knockout of NPM in cells and mice disturbed the genomic stability, which it contributed to growth-suppressing pathways through the interaction between NPM and ARF. So the loss of NPM expression could contribute to tumorigenesis [9].

Although NPM has a great diversity of biological functions, its physiological function in tumorigenesis is still a controversial issue on account of tumor suppressive and oncogenic functions of NPM. Due to the inconsistency of NPM functions, we preformed this meta-analysis to evaluate the prognostic value of NPM in patients with solid tumors. It expected NPM could serve as a novel biomarker for diagnosis and treatment in solid tumors.

## Methods

### Literature search and study selection

A comprehensive literature search was conducted by using the electronic databases PubMed, Web of Science, Embase, and China National Knowledge Infrastructure databases (up to October, 2017) with the following terms: “nucleophosmin or NPM or B23 or numatrin or NO38 or NPM1” and “cancer or tumor or carcinoma or malignancy or neoplasm” and “prognosis or prognostic or survival or mortality or outcome”. The results were restricted to human studies. We also searched the reference lists of the reviews on related topics to identify additional studies.

We diligently screened the eligible studies with the following inclusion criteria: (1) studies assessing the association of NPM expression and prognostic outcomes in solid tumors; (2) NPM expression has been measured in tumor tissue by immunohistochemistry (IHC) stain; (3) dividing NPM expression into “high” and “low” or “positive” and “negative”; (4) offering hazard ratios (HRs) with 95% confidence intervals (CIs) or sufficient information for estimating these statistics; (5) studies were written as full papers. We excluded the following studies: letters, reviews, abstracts, editorials, case reports, expert opinions, or animal experiments.

### Data extraction and quality assessment

All data included in this meta-analysis were reviewed and extracted independently by two investigators using a predefined form. The collected data included the first author name, publication year, study region, cancer type, number of patients, age, sex, cancer stage or grade, percentage of high NPM expression and the cutoff value, median follow-up months, HR and 95%CI of high NPM expression group versus low group. For studies that showed only Kaplan-Meier curves, we extracted the survival data by Engauge Digitizer (version 4.1). And the estimated HR and 95%CI were calculated using Tierney’s method [21].

The quality of each included study was carefully assessed by two independent authors using Newcastle-Ottawa Quality Assessment Scale (NOS) [22]. Three evaluation contents contained selection, comparability, and outcome of interest. The studies with higher than 6 scores were considered as high-quality studies.

### Statistical analysis

Statistical analyses were performed using STATA 14.0 software (Stata Corporation, College Station, TX, USA). The pooled HRs and 95%CIs were used to evaluate the relationship between NPM expression and patients’ overall survival (OS). Additionally, odds ratios (ORs) and their 95%CIs were used to assess the association between NPM expression and the clinicopathological features of solid tumors. The statistical heterogeneity was measured using the Cochran’s Q-test and I-squared test [23]. I^2^ > 50% or *P* < 0.10 were considered as significant heterogeneity. Publication bias was assessed using Begg’s funnel plot, the symmetry of funnel plot was evaluated by Egger’s test (*P* < 0.05 was considered as statistically significance) [24]. The sensitivity analysis was carried out by sequentially removing each study to evaluate the influence of single study on the pooled outcomes. All analysis were calculated using the random-effects model.

## Results

### Description of eligible studies

**Fig. 1:**
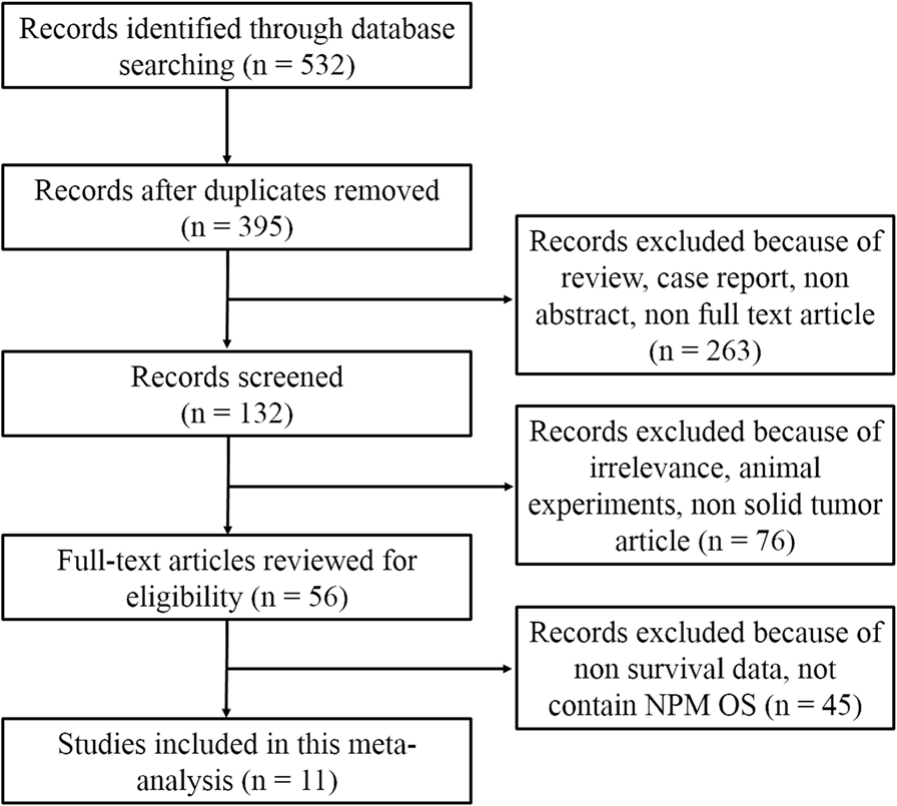
Flow diagram of the study selection process. NPM: nucleophosmin; OS: overall survival

Initially, a total of 532 studies were identified by electronic search in primary databases. Then 11 eligible studies were included in the final meta-analysis according to the inclusion and exclusion criteria. The concise process of literature selection was presented in Fig. 1.

All 11 studies with a total of 997 patients were used immunohistochemistry method to detect the expression of NPM. The patients from China [3, 14, 25–29], Japan [30], Taiwan [31, 32], Italy [15] were diagnosed with various tumors, including colon cancer, Ewing’s sarcoma, hepatocellular carcinoma, gastric cancer, ovarian serous cancer, colorectal carcinomas, glioma, astrocytoma, pancreatic ductal adenocarcinoma, bladder urothelial carcinoma. The main characteristics of these included studies are shown in Table 1. Of the 11 included studies, the median follow-up time ranged from 0.6 to 179 months, even 4 studies did not report it [3, 27, 31, 32]. One study [15] did not state the percentage of high NPM expression, and the cutoff value for defining positive or high NPM expression could be extracted from 9 studies. The HR and 95%CI for assessing the association of NPM expression and overall survival were directly reported in 5 studies, and those of other studies only showed Kaplan-Meier survival curves [3, 14, 28–30, 32]. All of included studies were high quality, and they got a score ≥ 6 NOS assessment.

**Table 1:** Characteristics of 11 eligible studies in this meta-analysis

Author	Year	Study region	Cancer type	NO. of patients	Age, median (range)	Male/ female	Cancer stage or grade	Percentage of high NPM Cutoff value	Follow-up months	HR and 95% CI	Quality score
Kazutaka Kikuta [30]	2009	Japan	Ewing’s sarcoma	34	20(1–63)	22/12	Stage I-III	23/34(68%), NA	57.5 (8–179)	NA	6
Yan Liu [3]	2012	China	Colon cancer	31	NA	13/18	NA	19/31(61%), H-score ≥ 3	NA	NA	9
Shao-Jung Lo [31]	2013	Taiwan	Hepatocellular carcinoma	110	NA	75/35	Stage I-IV	17/110(15%), score 3	NA	HR: 1.92 CI (0.92–4.02) *P* = 0.082	9
Yong Li [25]	2014	China	Gastric cancer	108	NA	76/32	Stage I-IV	57/108(53%), score 5–12	31(3–53)	HR: 1.970 CI (1.134–3.422) *P* = 0.0162	9
Ambrogio P. Londero [15]	2014	Italy	Ovarian serous cancer	73	NA	0/73	Stage I-IV	NA, H-score > 10	39(22–57)	HR: 2.98 CI (1.46–6.08) *P* < 0.05	7
Yu-Feng Yang [26]	2014	China	Colorectalcarcinomas	161	NA	90/71	Grade 1–3	104/161(64.6%), > 50%	49.24 (23–81)	HR: 1.919 CI (1.056–3.488) *P* = 0.032	9
Jian-Guo Chen [27]	2015	China	Glioma	90	NA	58/32	Grade II-IV	50/90(55.6%), H-score ≥ 5	NA	HR: 2.380 CI (1.149–4.929) *P* = 0.020	9
Yen-Hsin Kuo [32]	2015	Taiwan	Astrocytoma	99	NA	51/48	Grade I-IV	51/99(51.5%), > 50%	NA	NA	9
Yi Zhu [28]	2015	China	Pancreatic ductal adenocarcinoma	65	62(34–85)	40/25	Grade 1,2,4	46/65(69%), H-score ≥ 4	12(0.6–87)	NA	7
Fang Zhou [14]	2016	China	gastric cancer	131	63.5(28–85)	96/35	Stage I-III	86/131(66%), H-score ≥ 5	39(3–55)	NA	9
Hai-Ping Wang [29]	2017	China	Bladder urothelial carcinoma	95	NA	66/29	NA	67/95(70.5%), NA	81.5 (60–105)	NA	8

### The prognostic value of NPM in solid tumor patients’ overall survival

**Fig. 2:**
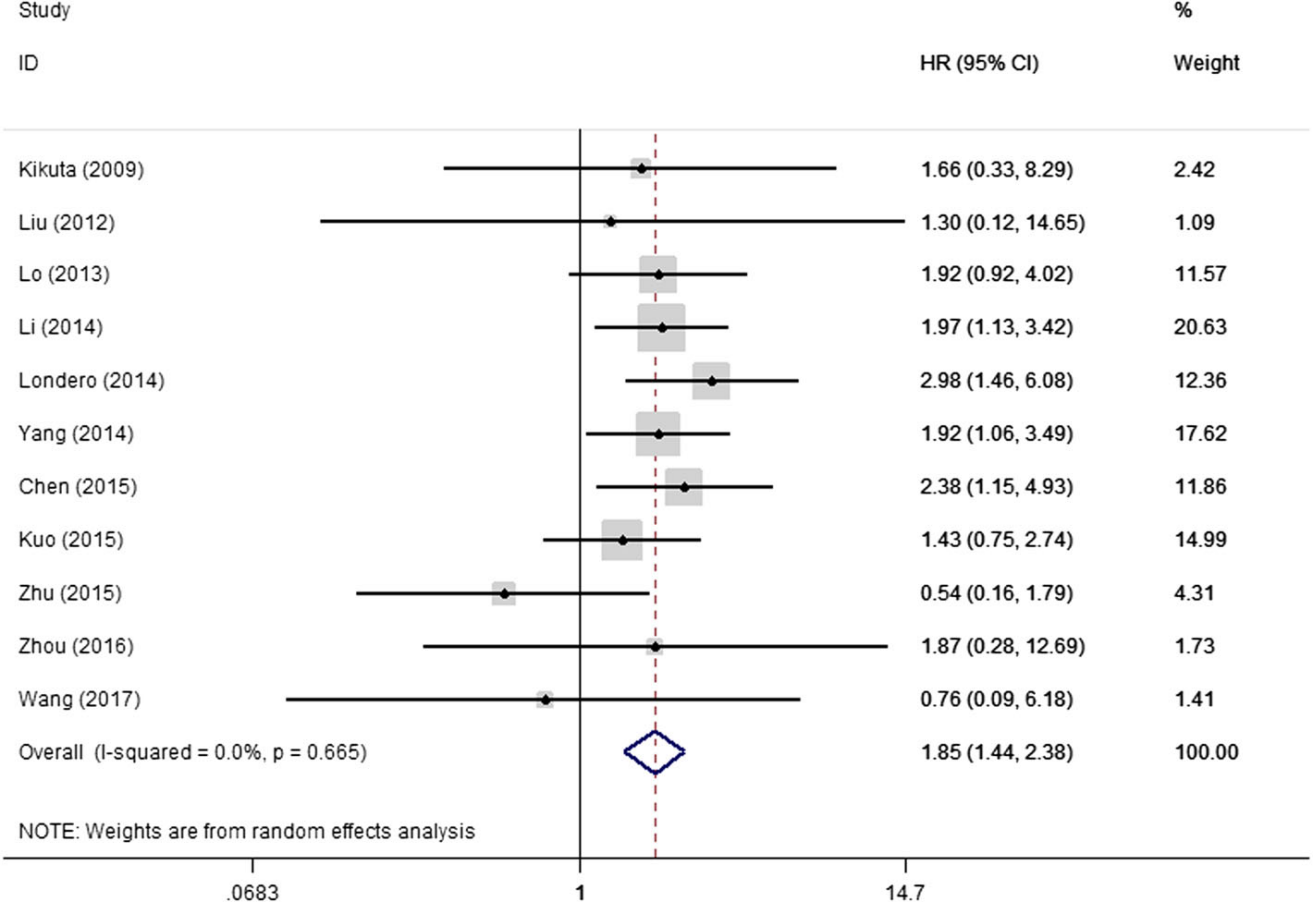
Forrest plots of studies assessing NPM expression and patients’ overall survival

All 11 studies were included in this meta-analysis of solid tumor patients’ overall survival. A random-effects model was used to calculate the pooled HR and 95% CI. The result demonstrated that the solid tumor patients with higher expression of NPM had poor prognosis (HR = 1.85; 95%CI: 1.44–2.38; *P* < 0.001). The heterogeneity test showed *P* value was 0.665 and I^2^ value was 0.0% (Fig. 2).

### Association of NPM and clinicopathological features

**Fig. 3:**
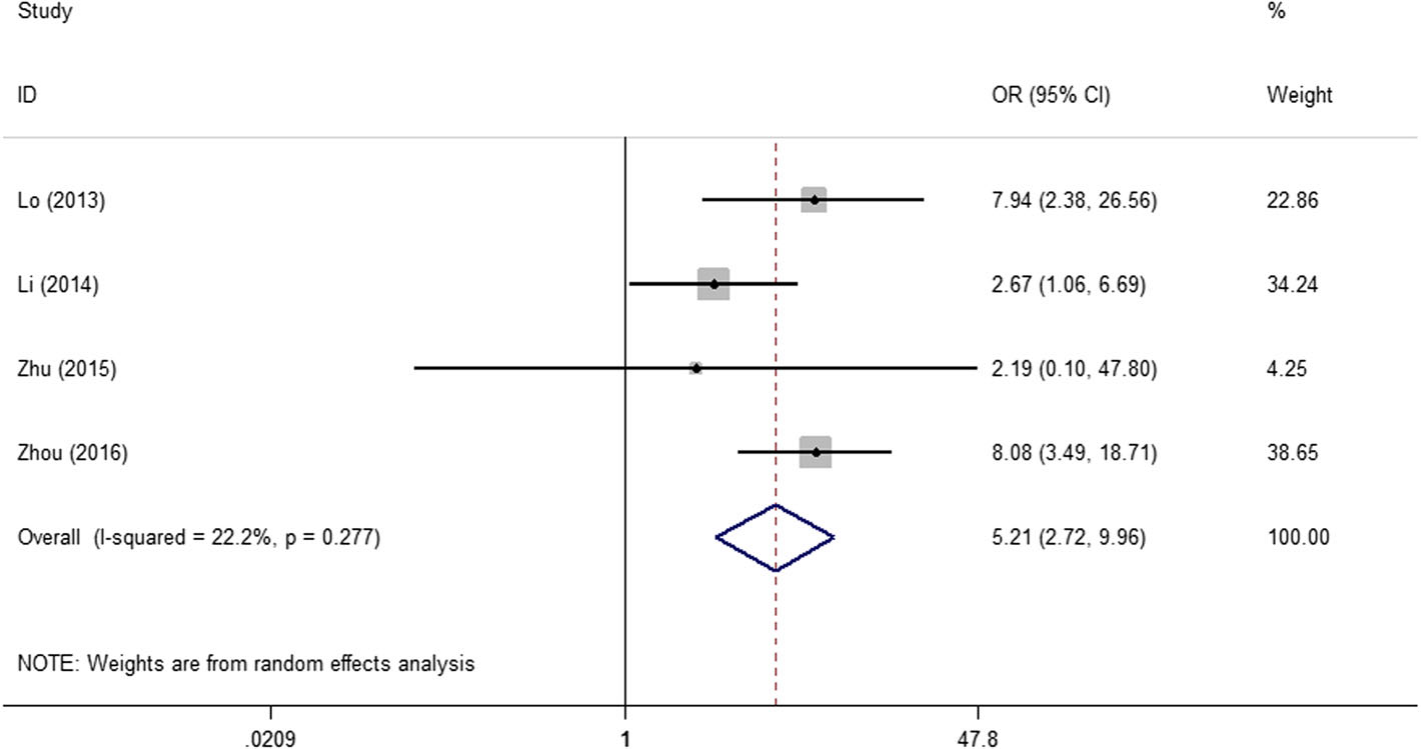
Forrest plots of studies evaluating NPM expression and tumor stage

**Fig. 4:**
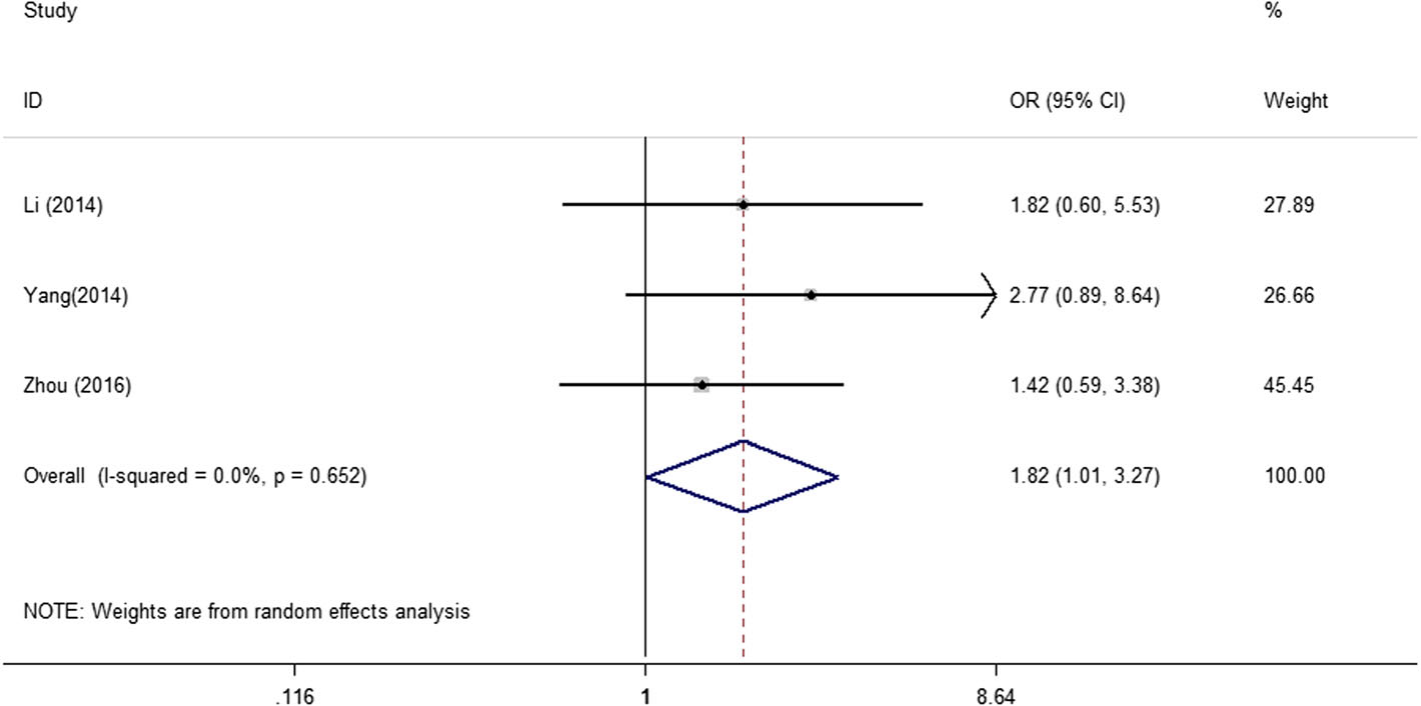
Forrest plots of studies evaluating NPM expression and differentiation grade

To explore the role of NPM expression in different solid tumors, we also investigated the correlation between NPM levels and clinicopathological features. The results illustrated in Table 2, NPM expression was not related with solid tumors patients’ age, gender and tumor size. However, positive or high expression of NPM was significantly associated with advanced tumor stage (4 studies; III/IV vs. I/II; pooled OR = 5.21; 95%CI: 2.72–9.96; *P* < 0.001; random effects) and advanced differentiation grade (3 studies; poor vs. well/moderate; pooled OR = 1.82; 95%CI: 1.01–3.27 *P* = 0.046; random effects) (Figs. 3 and 4).

**Table 2:** Meta-analysis of NPM expression and clinicopathological features in solid tumors

Categories	Studies	Pooled OR	95% CI	Heterogeneity I^2^(%)	*P* Value
Age(≥60 vs. < 60)	6	0.755	0.403–1.416	52.1	0.381
Gender (male vs. female)	9	0.741	0.550–1.000	0.0	0.050
Tumor size (≥4 cm vs. < 4 cm)	3	0.771	0.438–1.358	0.0	0.368
Tumor stage (III/IV vs. I/II)	4	5.209	2.724–9.959	22.2	< 0.001
Differentiation grade (poor vs. well/moderate)	4	1.817	1.010–3.266	0.0	0.046

### Sensitivity analysis

**Fig. 5:**
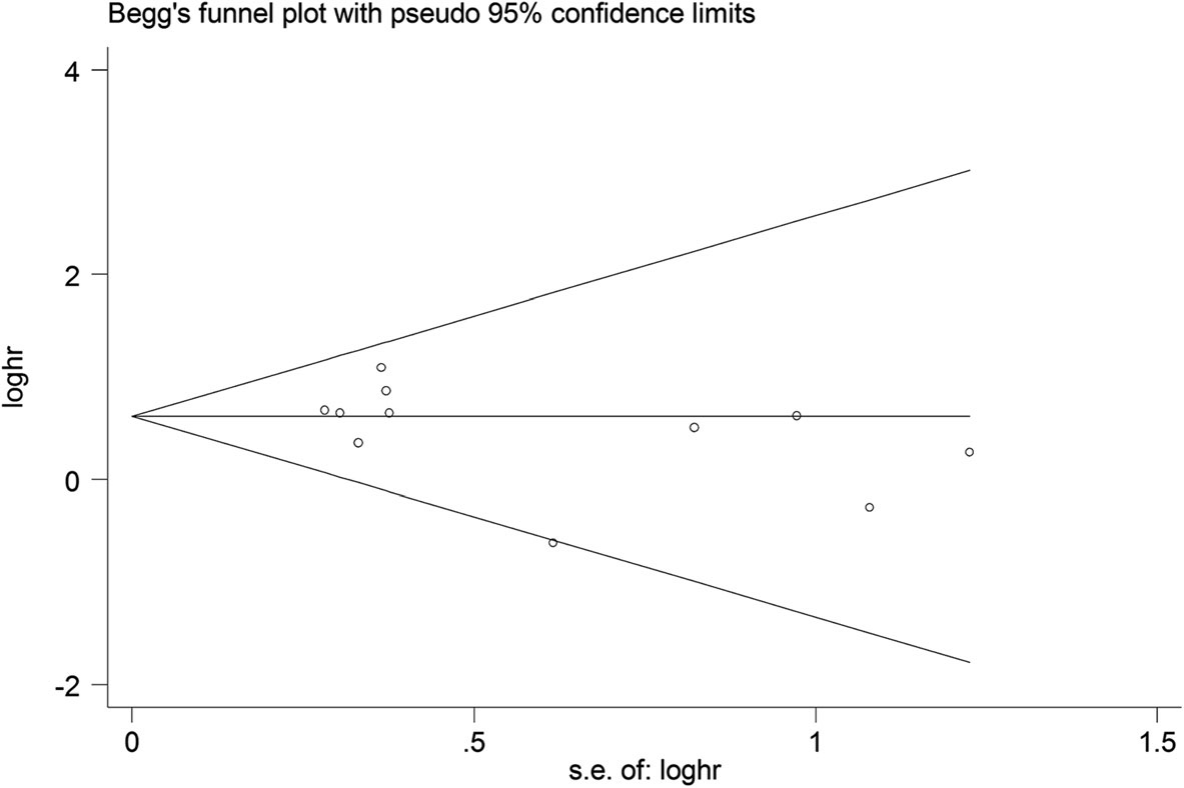
Sensitivity analysis of the meta-analysis

Sensitivity analysis was preformed to assess the potential heterogeneity of each study on the patients’ overall survival. The results suggested that the pooled HRs was not influenced the combined results after removing any individual study (Fig. 5). This indicated that the results of meta-analysis were stable and reliable.

### Publication bias

**Fig. 6:**
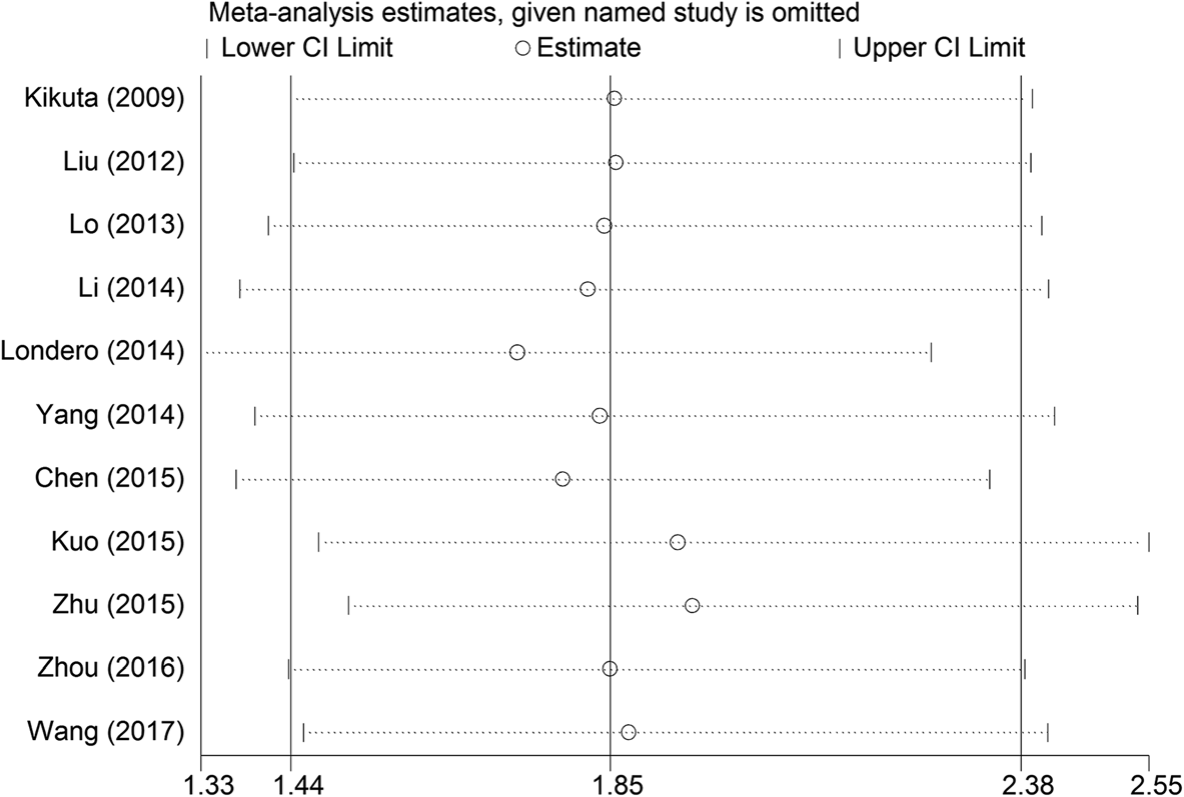
Begg’s funnel plot for potential publication bias of the included literatures

As shown in Fig. 6, the shape of the funnel plot for OS was symmetrical, and the results from Begg’s test (*P* = 0.119) and Egger’s test (*P* = 0.191) also revealed that there was no obvious publication bias in this meta-analysis.

## Discussion

As a multifunctional factor, NPM participated in cell growth, proliferation, transformation and apoptosis [9, 33]. In the past studies, most of researchers found that overexpression of NPM may promote tumors progression and predict poor prognosis of cancer patients, and they even expected NPM as a new biomolecular marker for improving clinical cancer therapy and outcomes [4, 32, 34]. However, the prognostic value of NPM among different solid tumors is still in contradiction. By summarizing the findings of published literatures, we conducted this comprehensive meta-analysis to assess the association between expression of NPM and the prognosis of solid tumor patients.

This meta-analysis included 11 studies with 997 patients, and the systematically evaluated outcomes demonstrated the high level of NPM was significantly correlated to poor overall survival in various solid tumors. It suggested that NPM overexpression was a potential independent predictor of poor prognosis in most solid tumors, including Ewing’s sarcoma, hepatocellular carcinoma, gastric cancer, ovarian serous cancer, colorectal carcinomas, glioma, astrocytoma, pancreatic adenocarcinoma and bladder carcinoma. Moreover, sensitivity analysis reinforced the reliability of this meta-analysis outcomes. And the publication bias was not detected in the pooled outcomes. Although four studies didn’t report the median follow-up time, we estimated the outcomes by Kaplan-Meier curves of overall survival, and they didn’t impact the stability and reliability of meta-analysis. Besides, according to the subgroup analyses, we also investigated the association between NPM expression and clinicopathological features. The results indicated that the high expression of NPM was obviously related to advanced tumor stage and advanced differentiation grade, which suggested that NPM level probably involved in tumor progression and then affected tumor patients’ overall survival.

It has been demonstrated that abnormal expression of NPM could promote tumorigenesis and tumor progression in more different cancers. For instance, as a critical regulator, NPM was overexpressed in prostate cancer, and it regulated cell proliferation [35]. The high expression of NPM is associated with local recurrence, and NPM might be used as a prognostic indicator in oral squamous cell carcinoma [36]. Moreover, NPM was overexpression in thyroid tumors, its dysregulation occurred at protein level and related to an increase of p-Akt level of transformed thyrocytes [16]. NPM might be a useful immunohistochemical marker for differential diagnosis between oncocytoma and chromophobe renal cell carcinomas (RCCs), and increased nucleolar NPM expression in RCCs appeared to be associated with tumor progression [37]. All these researches proved the significant value of NPM as a biomarker in the occurrence and progress of solid tumors. While the mechanism of NPM overexpression should still be further explored and investigated.

To our knowledge, several limitations may exist in our meta-analysis. Firstly, some of the studies did not report the HRs about NPM expression and OS, we only calculated them through Kaplan-Meier survival curves or univariate analysis. These may be less reliable than the accurate HRs directly obtained from published articles [38]. Secondly, the methods and cut-off values for assessing NPM expression and defining NPM positivity or high level were inconsistent. This may lead to heterogeneity. Thirdly, due to the limited number of studies, we were not able to conduct detail subgroup analyses to avoid the tumor heterogeneity. Fourthly, the follow-up period in all included studies were considerably different and some of them did not report it. In consequence, the further studies should need to explore the influence of these confounding factors on the pooled results.

## Conclusions

This present study is the first and comprehensive meta-analysis that illustrates the possible prognostic role of NPM up-regulation in solid tumors. Our results suggest that NPM may be a useful prognostic biomarker, and targeting NPM might be a promising therapeutic approach for solid tumors. But further data are still required for the potential effect of NPM on the different solid tumors from future researches.

## Abbreviations

CI: confidence intervals
HR: Hazard ratio
IHC: immunohistochemistry
NOS: Newcastle-Ottawa Quality Assessment Scale
NPM: Nucleophosmin
OR: Odds ratio
OS: Overall survival
